# Hailey–Hailey Disease: A Novel Method of Management by Radiofrequency Surgery

**DOI:** 10.4103/0974-2077.44167

**Published:** 2008

**Authors:** Nandini AS, Venkataram Mysore

**Affiliations:** *Venkat Charmalaya Centre for Advanced Dermatology, Subbanna Garden, Vijaynagar, Bangalore-560 040, Karnataka, India*

**Keywords:** Hailey–Hailey disease, radiofrequency, ablation

## Abstract

Hailey–Hailey disease is a chronic, recurrent disease that causes considerable morbidity to the patient. While the medical line of treatment is only palliative, different surgical modalities have been reported to offer longer lasting remission. We report a case of Hailey-Hailey disease successfully treated with radiofrequency surgery.

## INTRODUCTION

Hailey-Hailey disease is an autosomal dominant genodermatosis characterised by recurrent vesicles and erosions usually affecting the flexural areas such as neck, axillae and groins.[1] Medical treatment[1–3] consists of topical steroids, antibiotics, tacrolimus and others. However, it provides only short-term relief, with lesions recurring frequently and is therefore unsatisfactory. Several surgical modalities have been reported to be effective, including excision, excision with grafting, dermabrasion, carbon dioxide laser and Er:YAG laser[1]. We hereby report a case of Hailey–Hailey disease successfully treated with radiofrequency ablation.

## CASE REPORT

**Figure 1:**
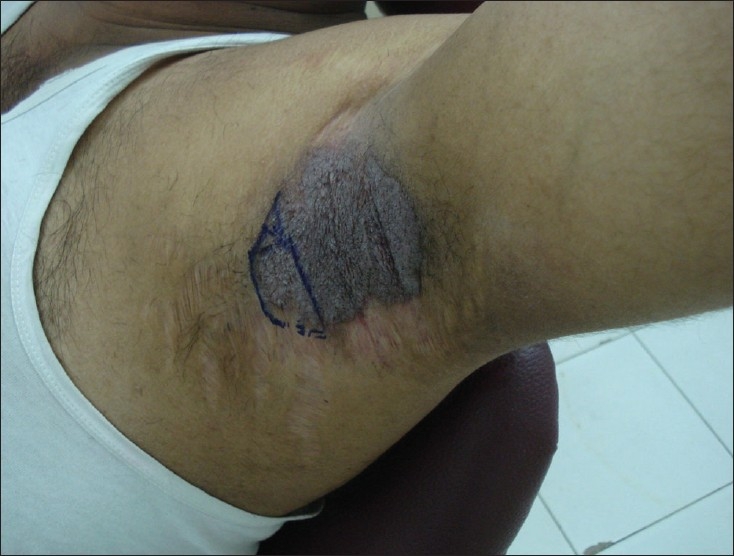
Hailey–Hailey disease, left axilla showing erosions and plaques

A 35-year-old male patient presented with a 20-year history of recurrent macerated, foul smelling, pruritic, erythematous erosions on both axillae [Figure 1]. Family history revealed that the patient’;s paternal uncle also had similar lesions. Patient had been treated with many topical medications including potent topical steroids, which had resulted in side effects such as extensive striae in and around the axillae. However, the topical medications provided only mild and temporary relief. Biopsy was performed, which confirmed the diagnosis of Hailey–Hailey disease.

**Figure 2:**
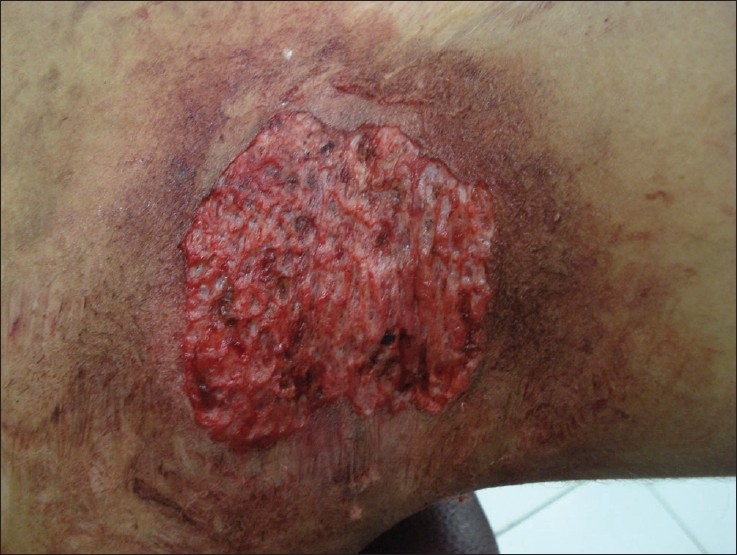
Lesion immediately after radiofrequency

**Figure 3:**
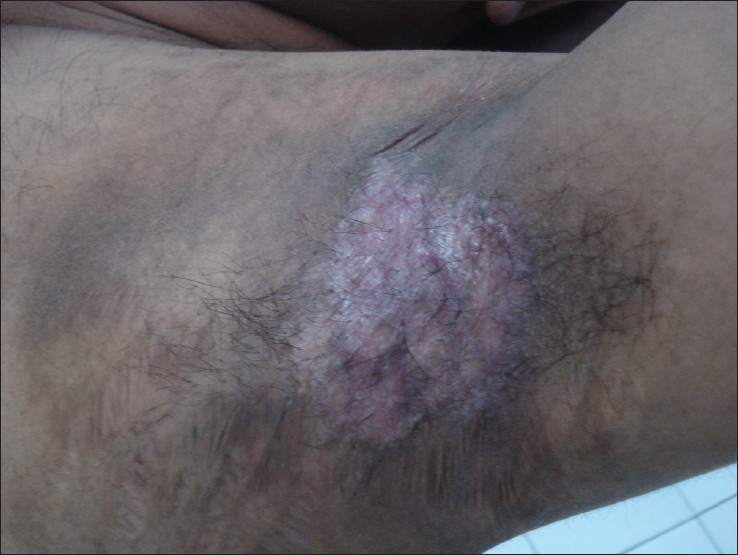
Left axilla lesion completely healed with scarring, 16 weeks after treatment

Since medical treatments had been ineffective, it was decided to consider ablative surgical treatment using radiofrequency as it was cheap and could be easily administered. Patient was asked to stop all topical steroids on the left axilla and under antibiotic coverage, radiofrequency ablation using Ellman Surgitron™, model – FFDF.EMC (New York, NY, USA). It generates a 3.8 MHz frequency with a peak power output of 140 Watt ± 20% in continuous mode. A loop probe was used to remove the entire plaque upto the level of mid-dermis [Figure 2] in the left axilla, so as to cause scarring. The right axilla was treated with only topical steroid–antibiotic combination and served as a control. The side treated by radiofrequency showed marked and rapid improvement and complete clearance of the lesions and absence of relapse over a follow-up period of 16 weeks [Figure 3]. During the same period the right axilla, which continued to receive local steroid–antibiotic combination creams, showed several recurrent episodes of the disease over the same period. This ruled out the possibility of the result of the test side being attributable to spontaneous improvement in the course of the disease. The patient was eager to get a similar procedure on the right side and the same is now being planned.

## DISCUSSION

Hailey–Hailey disease is a rare autosomal dominant intraepidermal blistering disorder that is characterised by mutations in the gene that encodes golgi-associated Ca^2+^ ATPase (ATP2c1) leading to abnormal intracellular Ca^2+^ signalling, resulting in acantholysis in stratum spinosum.[1]

The condition usually presents in the third or fourth decade, with flaccid vesiculopustules, crusted erosions or expanding circinate plaques in areas of friction such as neck, axilla, groins and perineum. Flexural lesions may be hypertrophic and malodorous with soft, flat, moist vegetation and fissures.[2]

Medical line of therapy[1–3] includes topical therapy like corticosteroids, topical antimicrobials, cyclosporine, tacalcitol, tacrolimus and, more recently, botulinum toxin. Systemic therapy with antimicrobials, retinoids and other immunosuppressive drugs has been tried, but the result with these treatments is not long lasting.[1,3]

Only ablative methods have been reported to lead to long-term remission of the lesions in Hailey–Hailey disease. The different surgical methods reported so far include excision/skin grafting,[1] dermabrasion,[4] CO_2_ laser[4,5] and ER:YAG laser.[6] Literature search on Pubmed using the key words radiofrequency, and Hailey–Hailey disease did not reveal any publication regarding the use of radiofrequency in the treatment of the condition. We feel, therefore, that this is possibly the first report of the use of radiofrequency surgery in this condition.

Radiofrequency surgery is an effective, simple and cost-effective procedure. Like the other surgical techniques mentioned above, radiofrequency acts by causing ablation of the affected skin. The possible underlying mechanism for the efficacy of ablative methods is not fully known. Reepidermisation with normal keratinocytes produced from appendices, which do not express the molecular defect and the constitution of dermal cicatricial tissue are the two currently proposed hypotheses.[5]

We are aware of the fact that the short duration of follow-up in this case report is a limitation. However, the beneficial effect obtained so far is encouraging, particularly because radiofrequency is a cheap and easy mode of treatment. Studies with longer follow-ups will serve to establish the role of radiofrequency for the management of this disease.
